# Sex hormones and oesophageal adenocarcinoma: influence of childbearing?

**DOI:** 10.1038/sj.bjc.6602810

**Published:** 2005-09-27

**Authors:** J Lagergren, C Jansson

**Affiliations:** 1 Unit of Esophageal and Gastric Research, Department of Molecular Medicine and Surgery, Karolinska Institutet, Stockholm, Sweden; 2 Department of Medical Epidemiology and Biostatistics, Karolinska Institutet, Stockholm, Sweden

**Keywords:** fertility, sex hormones, oestrogen, cardia, neoplasm

## Abstract

The male predominance of oesophageal adenocarcinoma might be explained by oestrogen protection in women. If true, female patients might have sex hormonal disturbances rendering impaired fertility. The influence of childbearing on the risk of oesophageal adenocarcinoma was investigated in a Swedish population-based case (*n*=63) -control (*n*=141) study. Childless women were not at increased risk compared to childbearing (OR=0.82; 95% CI=0.25–2.72), as neither were women with 0–1 children compared to women with at least three children (OR=0.93; 95% CI=0.35–2.49). In conclusion, we found no inverse assciation between childbearing and oesophageal adenocarcinoma.

The striking male predominance among patients with adenocarcinoma of the oesophagus and gastro-oesophageal junction (cardia) remains entirely unexplained (Lagergren, 2005; Wong and Fitzgerald, 2005). Detection of the cause of this sex imbalance might also be a key to explaining the alarming rise in incidence of these tumours in Western societies (Lagergren, 2005; Wong and Fitzgerald, 2005). The exposure to the main established risk factors, gastro-oesophageal reflux and obesity, is evenly distributed between the sexes and should therefore not be responsible for the sex difference (Lagergren, 2005; Wong and Fitzgerald, 2005). A critical role of sex hormones has been suggested, but never proven (Lagergren and Nyren, 1998). The rarity of these tumours among women has prohibited valid research regarding this issue. Oesophageal adenocarcinomas often express oestrogen receptors, which suggests biological involvement of steroid hormones (Akgun *et al*, 2002). We hypothesised that women are effectively protected against development of oesophageal and cardia adenocarcinoma through their normal levels of oestrogens, and that the few women who develop these tumours might often have sex hormonal disturbances that could impair their fertility. If this is true, childbearing would be a marker of a decreased risk of adenocarcinoma of the oesophagus or cardia. This hypothesis has not to our knowledge been evaluated previously. We therefore analysed the relation between childbearing and risk of oesophageal and cardia adenocarcinoma in a Swedish nationwide, population-based, case–control study.

## METHODS

The design and organisation of our case–control study has been described in detail elsewhere (Lagergren *et al*, 1999). The current study comprised only female Swedish residents of ages below 80 years with oesophageal (number=24) or cardia (number=39) adenocarcinoma diagnosed during the 3-year period 1995 through 1997. To reduce tumour misclassification, uniform and thorough documentation of the tumours was introduced at the 195 participating departments throughout Sweden, and finally, one pathologist reviewed the histological slides of virtually all (97%) patients. Female control participants (number=141) were randomly selected from the computerised Total Population Register of Sweden and frequency-matched to the age distribution of the case patients. All study participants underwent computer-aided face-to-face interviews by professional interviewers that included questions about childbearing, age, reflux symptoms (categorised as yes or no), body mass index (in quartiles among the control participants), tobacco smoking status (never, previous and current, assessed 2 years before the interview), alcohol use (in four groups), socioeconomic status (in six groups), living with a partner (or alone), and intake of fruit and vegetables (in three groups). Using conditional logistic regression, relative risks were estimated by odds ratios with 95% confidence intervals (CI). Potential confounding by the covariates listed above was adjusted for in multivariable analyses. Individual informed consent was obtained from all study participants, and all regional ethics committees in Sweden approved the study.

## RESULTS

In total, 63 women with oesophageal or cardia adenocarcinoma (85% of all eligible) and 141 female control persons (73% of all eligible) participated. Reasons for nonparticipation among cases were mainly physical or mental disorders or early death, while most nonparticipating controls declined to participate. Some characteristics of the case and control participants are presented in Table 1. Reflux symptoms, overweight, smoking, and manual workers were more common among case patients than among controls, while high alcohol consumption and living alone were evenly distributed. Data on childbearing are presented in Table 2. The childbearing frequencies were the same between cases and controls (78% in both groups). Women who had never given birth were not at an increased risk of oesophageal or cardia adenocarcinoma compared to women who had given birth (adjusted odds ratio 0.82; 95% CI 0.25–2.73). No association was found between number of children born and risk of these tumours (Table 2). Women who had delivered no children or one child were not at higher risk than those who had given birth to three children or more (adjusted odds ratio 0.93; 95% CI 0.35–2.49). Neither age at first delivery nor time between first and last delivery influenced the risk of oesophageal or cardia adenocarcinoma (Table 2). As shown in Table 2, the multivariable adjusted odds ratios were generally similar to the crude risk estimates, indicating lack of strong confounding by the included covariates listed in the Methods section.

## DISCUSSION

This study did not supply any evidence of a reduced risk of oesophageal or cardia adenocarcinoma related to fertility as measured by childbearing.

Although we included virtually all female cases of oesophageal and cardia adenocarcinomas throughout Sweden during a 3-year period, and we combined these tumour sites into one, the sample size was too limited to rule out moderate associations. The point estimates were all confidently close to unity, however, which supports our interpretation that childbearing does not strongly reduce the risk of developing these tumours. Moreover, if our hypothesis was true, we would expect strong effects that should be possible to reveal in the study. The similar sex ratios of oesophageal and cardia adenocarcinomas justified combining these tumours in the analyses. Moreover, their risk factor profiles are similar, although not identical (Lagergren, 2005; Wong and Fitzgerald, 2005). Separate analyses of these two tumour sites revealed no strong difference, but the statistical power in these comparisons was low (data not shown). Advantages of our study include the truly population-based design with strict sampling of control participants, the prospective and rapid case ascertainment with high participation frequencies, the personal interviews with all study participants, allowing robust exposure assessment, including all plausible confounding factors, and the prospective and thorough tumour classification.

The rarity of oesophageal and cardia adenocarcinoma among women has virtually prohibited epidemiological research of the risk factors for these tumours among females separately. In the absence of known risk exposures that are sufficiently skewed between the sexes, sex hormonal influence has been hypothesised as playing a key role (Lagergren and Nyren, 1998). However, previous epidemiological studies of sex hormones are sparse. In a cohort study of oestrogen-treated male prostate cancer patients, we found no reduced risk of oesophageal adenocarcinoma (Lagergren and Nyren, 1998), a finding that contradicts sex hormonal influence and is hence in line with the results of the current study. On the other hand, in a case–control study among females in the United Kingdom, breastfeeding was associated with a 60% reduced risk of oesophageal adenocarcinoma (Cheng *et al*, 2000), a finding that suggests sex hormonal influence. Obviously, more research is needed.

In conclusion, our hypothesis of an inverse relation between childbearing and risk of oesophageal or cardia adenocarcinoma was not supported in this study. However, this finding should not discourage further research of sex hormones in the aetiology of these tumours. A role for sex hormones cannot be excluded on the basis of the available data. The mystery of the male predominance among patients with oesophageal and cardia adenocarcinoma needs to be solved in future research.

## Figures and Tables

**Table 1:** Selected characteristics of the female case patients and control participants

	**Control participants**	**Oesophageal or cardia adenocarcinoma**
**Variable**	**Number (%)**	**Number (%)**
Total	141 (100)	63 (100)
Age < 60 years^a^	107 (76)	49 (78)
Reflux^b^	17 (12)	25 (40)
Overweight/obesity^c^	36 (26)	32 (51)
Ever smokers^d^	51 (36)	34 (54)
High alcohol intake^e^	7 (5)	2 (3)
Living alone^f^	9 (6)	4 (6)
Manual workers^g^	56 (40)	37 (59)

a The study participants were frequency-matched by age.

b Symptoms of reflux occurring at least once weekly at least 5 years before interview.

c Body mass index (kg m^−2^)>25 kg m^−2^, 20 years before interview.

d Ever regular tobacco smoker (previous or current smoker, 2 years before interview).

e At least 70 g per week of pure alcohol, 20 years before interview.

f Not having lived with a partner (married/cohabitant) for at least 1 year.

g Socioeconomic status of longest duration, derived from lifetime occupational histories.

**Table 2:** Childbearing and risk of oesophageal and cardia adenocarcinoma among female study participants

	**Control participants**	**Oesophageal or cardia adenocarcinoma**		
**Variable**	**Number (%)^a^**	**Number (%)^a^**	**Crude OR^b^ (95% CI)**	**Adjusted OR^c^ (95% CI)**
*Children*				
Yes	110 (78)	49 (78)	1.00 (reference)	1.00 (reference)
No	17 (13)	8 (13)	1.07 (0.43–2.65)	0.82 (0.25–2.73)

*Number of children born* d				
⩾3	38 (27)	17 (27)	1.00 (reference)	1.00 (reference)
2	49 (35)	20 (32)	0.92 (0.42–2.00)	1.26 (0.48–3.32)
0–1	40 (28)	20 (32)	1.09 (0.50–2.39)	0.93 (0.35–2.49)

*Age at first birth* d				
16–22 years	34 (24)	19 (30)	1.00 (reference)	1.00 (reference)
23–25 years	33 (23)	12 (19)	0.65 (0.28–1.53)	1.07 (0.36–3.15)
26–39 years	43 (30)	18 (29)	0.75 (0.34–1.64)	1.12 (0.41–3.05)

*Years between first and last birth* d ^,^ e				
8–22 years	29 (21)	15 (24)	1.00 (reference)	1.00 (reference)
4–7 years	34 (24)	11 (17)	0.66 (0.26–1.65)	0.43 (0.13–1.43)
1–3 years	22 (16)	11 (17)	1.12 (0.41–3.05)	1.17 (0.32–4.19)

a Persons with not applicable or missing data in any covariate included in any of the models were excluded. For example, in the analysis of years between first and last birth, persons with no or one children were excluded.

b Crude odds ratios controlled for age by matching.

c Odds ratios adjusted for reflux symptoms, body mass index, smoking, alcohol use, intake of fruit and vegetables, living with a partner and socioeconomic status, and controlled for age by matching. Years between first and last birth were not adjusted for living with a partner, since all cases and controls were married or cohabitant.

d The categories of age at first birth and years between first and last birth are based on tertiles among the controls.

e Among women who had given birth to at least two children.
